# Variation in antibiotic resistance patterns for children and adults treated at 166 non-affiliated US facilities using EHR data

**DOI:** 10.1093/jacamr/dlac128

**Published:** 2023-01-02

**Authors:** Shivani Sivasankar, Jennifer L Goldman, Mark A Hoffman

**Affiliations:** Department of Pediatrics, Children’s Mercy Hospital, Kansas City, MO, USA; Department of Pediatrics, Children’s Mercy Hospital, Kansas City, MO, USA; School of Medicine, University of Missouri-Kansas City, Kansas City, MO, USA; Department of Pediatrics, Children’s Mercy Hospital, Kansas City, MO, USA; School of Medicine, University of Missouri-Kansas City, Kansas City, MO, USA

## Abstract

**Background:**

Antibiotic resistance (AR) is a global public health threat. Surveillance of baseline AR and trends and emerging resistance among priority bacterial isolates with respect to the age of the patients and the type of healthcare setting are required due to differences in antimicrobial need and use in these populations.

**Methods:**

We performed a retrospective study using deidentified electronic health record (EHR) data in the Cerner Health Facts™ data warehouse. Antibiotic susceptibility data were extracted for all bacterial isolates of interest at 166 non-affiliated healthcare facilities reporting microbiology susceptibility results of the FDA recommended antibiotics between the years 2012 to 2017. We assessed and visualized the slope coefficient from linear regression to compare changes in resistance over time for the four patient care groups.

**Results:**

The trends in resistance rates to clinically relevant antibiotics were influenced by age and care setting. For example, ertapenem-resistant *Enterobacter cloacae* isolates from children overall increased significantly compared with adults (0.7% to 9.8%, 2.1% to 2.8%, *P* = 0.00013) and isolates from children in paediatric facilities increased significantly compared with facilities treating adults and children (0.1% to 27.1%, 0.9% to 3.8%, *P* = 0.0002).

**Conclusions:**

Large-scale analysis of EHR data from 166 facilities shows that AR patterns for some bug-drug combinations vary by care setting and patient age. We describe novel data visualizations to interpret large-scale EHR data on the prevalence and trends of AR that should influence antimicrobial prescribing and antimicrobial stewardship programme interventions.

## Introduction

The emergence of antibiotic-resistant (AR) bacteria endangers the efficacy of antibiotics and is a global public health crisis.^1,2^ The CDC estimates that 2.8 million people in the USA are infected each year with bacteria resistant to antibiotics, with an average of 35 000 deaths.^3^ AR infections cause significant morbidity and mortality worldwide and could reach up to 10 million deaths by 2050.^4,5^ AR infections can double the duration of hospital stays, increase mortality rate, prolong treatment and increase healthcare costs.^6,7^ US estimates suggest AR infections contribute $35 billion to healthcare costs per year.^8^

The CDC and WHO provide prioritized lists of AR bacteria based on level of concern to human health.^3,9^ AR variants of the ESKAPE pathogens (*Enterococcus faecium*, *Staphylococcus aureus*, *Klebsiella pneumoniae*, *Acinetobacter baumannii*, *Pseudomonas aeruginosa* and *Enterobacter* spp.) are considered to be emergent threats.^10,11^ Several US studies show an alarming increase in resistance among pathogenic Gram-negative bacilli, including *P. aeruginosa*, *A. baumannii*, *Escherichia coli*, *Proteus mirabilis*, *Serratia marcescens*, *Haemophilus* spp., *Klebsiella* spp., *Salmonella* spp., *Citrobacter* spp., *Enterobacter* spp. and *Shigella* spp.^12–20^ Gram-positive bacteria cause serious and difficult-to-treat infections, exacerbated by marked increases in AR among these bacteria, most notably MRSA, decreased susceptibility to penicillin in *Streptococcus pneumoniae* and vancomycin-resistant enterococci (VRE).^21–23^ Tracking emerging patterns of AR allows providers to precisely tailor antibiotic therapy and prevent the spread of existing AR.

Until recently, trends in drug-resistant infections in children have been relatively uncharacterized. Isolates from children have different selective pressure due to their immature immune system, somewhat distinct set of antibiotics or overall antimicrobial exposure.^24–27^ A study found a 700% surge in paediatric infections caused by the enteric pathogens resistant to multiple antibiotics in the USA over a period of 8 years.^28^ The type of facility (stand-alone paediatric or blended facilities caring for adults and children) could also be a factor for different resistance patterns in children, especially in hospital-acquired infections (HAIs).^29^ Paediatric facilities have been found to vary substantially in their use of antibiotics.^30^ AR patterns for priority pathogens have not been compared between children and adults nor between types of facility. This would inform paediatric care setting-focused antibiotic stewardship programmes (ASPs).

Data derived from electronic health records (EHRs) loaded into multi-institutional data warehouses provide a powerful resource for examining these issues. One such data resource, Cerner Health Facts™ (HF), contains microsusceptibility test results from multiple sites.^31^ The objective of this study was to compare the trends in resistance for the priority bacterial pathogens between children and adults as well as among children treated in primarily paediatric facilities and blended facilities.

## Materials and methods

### Data source

We derived the study data from Cerner HF database (Kansas City, MO, USA) populated by the daily extraction of discrete EHR data from participating organizations. HF data are deidentified in a manner compliant with US Health Insurance Portability and Accountability Act standards. In the 2018 version of the HF, 416 facilities associated with 84 non-affiliated health systems had contributed 5 million distinct microsusceptibility encounter data from 3 million patients to HF at intervals from January 2000 through 2017. Cerner ceased populating HF in mid-2018; the replacement, Cerner Real World Data, does not yet include discrete microbiology results. Between 2007 and 2012 Cerner implemented a variety of initiatives to improve the HF data quality and consistency. HF includes microbiology results, patient demographics, diagnoses, medication orders, other laboratory tests and clinical procedures. The Children’s Mercy Office of Research Integrity has designated research with HF data as ‘non-human subjects research’.

### Data definition

The efficacy of the antibiotics against the isolates is interpreted as resistant (R) if the bacteria are not inhibited by the recommended dosage of antibiotic and there is growth in the presence of the antibiotic; susceptible (S) if the isolates are inhibited by the recommended dosage of antibiotic; and intermediate (I) if there is limited growth in the presence of dilute antibiotics.^32^ The calculation of antimicrobial resistance (proportion of R relative of the total) is dependent on two prerequisites: the data should only consist of first isolates (the isolate of a bacterial species found first in a patient per encounter) and a minimum required number of 30 isolates per year for every group.^33,34^

### Data validation

Every encounter in HF is associated with a health system ID. Children’s Mercy Hospital (CMH) is a contributor to HF. In order to test the reliability of HF data, we extracted the 2017 CMH antimicrobial susceptibility data using the health system ID in HF and compared it with the CMH 2017 antibiogram (Table S1, available as Supplementary data at *JAC* Online).^35^ The antibiogram is populated annually by the CMH Clinical Microbiology laboratory. We evaluated the reliability of the data using a single-measurement, absolute-agreement, two-way, mixed-effects intraclass correlation (ICC) method.^36^

### Isolate-antibiotic combinations

Each record in the dataset includes the isolate, susceptibility test results, specimen source, and the time stamp of the test result verification. In order to minimize the number of isolates, we selected relevant pathogens based on the CDC report on greatest AR threats and the WHO list of global priority AR bacteria.^3,9^ There are 22 such organisms: *A. baumannii*, *Citrobacter freundii*, *Enterobacter aerogenes*, *Enterobacter cloacae*, *E. faecium, E. coli*, *Haemophilus influenzae*, *Klebsiella oxytoca*, *K. pneumoniae,* MRSA, MSSA, *P. mirabilis*, *P. aeruginosa, Salmonella* spp., *S. marcescens*, *Shigella* spp., CoNS, *Streptococcus* Group A, *Streptococcus* Group B, *S. pneumoniae*, *Streptococcus viridans* and VRE. In order to focus on clinically relevant antibiotics, we included CLSI recommended antimicrobial agents approved by the FDA for clinical use that are considered for routine testing and reporting by microbiology laboratories in the USA.^37^ There are 41 clinically relevant antibiotics for those 22 isolates (Table S2). Antibiotics primarily used to treat urinary tract infections are only considered for isolates whose source of infection is specifically urinary.

### Study design

**Figure 1.**
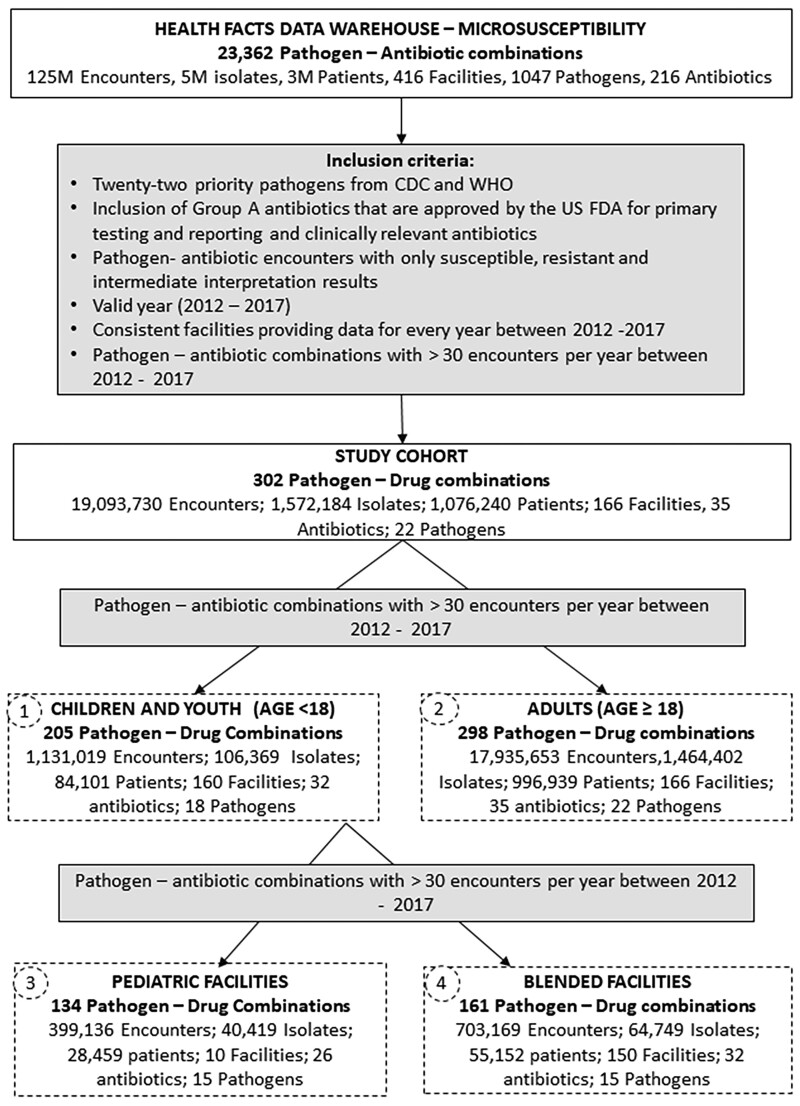
Data extraction and study design.

We conducted a retrospective study of encounters with the 22 priority bacteria with microbiology susceptibility results for the FDA recommended and clinically relevant antibiotics for the years 2012 to 2017 (Figure 1). *S. aureus* isolates were classified as MRSA if oxacillin/methicillin resistant, and MSSA if oxacillin/methicillin susceptible. We included encounters of the pathogens that reported valid susceptible, intermediate or resistant results with a valid date stamp. Facilities consistently reporting the microbiology susceptibility every year between the years 2012 and 2017 were included (166 facilities) whereas 250 facilities that did not fit the inclusion criteria were excluded. Facilities where the mean age of patients is less than 18 years were identified as likely paediatric facilities, and facilities treating both adults and children were identified as blended facilities. We separated the data cohort into four groups: encounters of the isolates from children (Age <18) (Group 1) and adults (Age >18) (Group 2); isolates of children from paediatric facilities (Group 3) and isolates of children from blended facilities (Group 4). Isolate-antibiotic combinations with the minimum required number of 30 isolates per year were included in every group (Figure 1).

### Analysis

We examined the resistant percentage of each pathogen-antibiotic pair individually and in relation to the four cohorts. We calculated the slope coefficient for the trend in proportion of resistance of every pathogen-antibiotic combination over the years 2012 to 2017 by utilizing linear regression. The data are installed in Microsoft Azure (Microsoft Corp., Redmond, WA, USA) and queries are performed with R Studio version 1.1.453 with R version 3.6.1. An analysis that we refer to as the multiple categorical slope (MCS) was performed for the total cohort and for all the four groups to identify significant antibiotic-resistant pathogens that are increased/decreased over the years. In the MCS plot, the calculated slopes of the pairs are plotted on the *y*-axis and similar to a Manhattan plot, the *x*-axis of the plot shows antibiotics as dots organized by pathogens, in different colored blocks. The pairs that increased during the period evaluated are indicated above the upper limit (95th percentile value of the slope) horizonal line whereas the pairs that decreased are indicated below the baseline lower limit (fifth percentile value of the slope). Total number of encounters per pathogen-antibiotic combination was included in the model as a covariate to address the bias of varying number of encounters. We compared the significance of the difference in the slope between children versus adult (Group 1 versus 2) as well as children in paediatric facilities versus blended facilities (Group 3 versus 4) through the comparison-MCS (C-MCS) plot. In the C-MCS plot, the effect of the interaction term (group × year) is plotted for every pathogen-antibiotic pair. The significance of the interaction term was tested to determine pathogen-antibiotic pairs with unequal slopes that represent a different pattern in the trend in resistance between children and adults as well as between children in paediatric facilities and children in blended facilities.

## Results

### Characteristics of data

The reliability of the data evaluated using ICC indicate that the agreement of the data between HF and the CMH antibiogram is considered to be excellent (ICC = 0.84; 95% CI: 0.77–0.88; after excluding outliers: ICC = 0.984; 95% CI: 0.977–0.99).

The 2018 version of the HF microbiology susceptibility data includes 125 million susceptibility results evaluated from 1047 pathogens screened for resistance to 216 antimicrobials resulting in 23 362 unique pathogen-antibiotic combinations. Isolate samples were reported from 550 distinct body source sites. Urinary tract isolates constituted 63% of all isolates. The majority of the susceptibility tests used the MIC or Kirby–Bauer method. Inclusion of 22 priority organisms and clinically relevant antibiotics resulted in 302 isolate-antibiotic combinations yielding 19 million encounters in the study cohort. This cohort consists of 1.5 million isolates identified from 1 million patients associated with 166 US facilities. Most (63%) facilities were non-teaching institutions, and the majority (80%) were located in urban environments. More than two-thirds (68%) had fewer than 200 beds, including 40 facilities with fewer than 5 beds, a marker for exclusively outpatient care. We noted that 28% of the facilities with patients in the cohort had 200–500 beds and 4% had more than 500 beds. There was an even distribution of the facilities in the South (*n* = 54), West (*n* = 45) and Midwest (*n* = 50) census regions whereas there were fewer facilities in the Northeast region (*n* = 17). We did not evaluate resistance patterns by patient geography.

There were 84 101 patients in the cohort younger than 18 years (13% of the HF cohort) whose encounters were segregated into Group 1 (children), with the remaining encounters in Group 2 (adults). Group 1 includes encounters from 160 facilities with 10 paediatric facilities. Those encounters were separated into Group 3 (paediatric facilities) whereas the remaining encounters are Group 4 (blended facilities). Data characteristics of these groups are summarized in Figure 1.

### Baseline resistance

**Figure 2.**
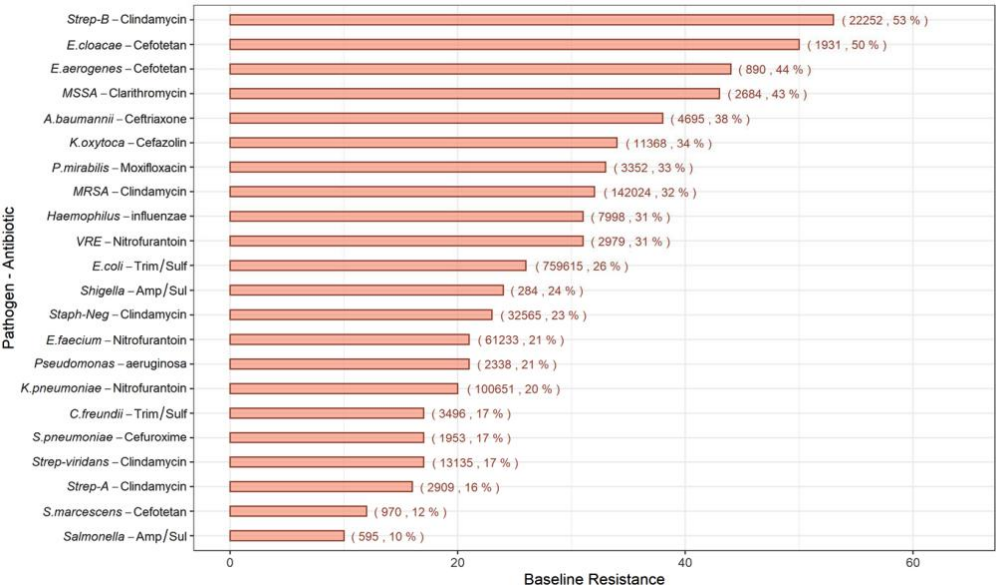
Baseline resistance of the study cohort (Total no. of resistant encounters, Resistance %).

Within the total study cohort, the 22 priority pathogens with the highest percentage of resistance to the clinically relevant antibiotics are shown in Figure 2 (number of isolates tested; percentage resistant). Among the pathogens with highest total level of resistance, *Streptococcus* Group B had the highest resistance to clindamycin (22 252; 52.79%).

**Figure 3.**
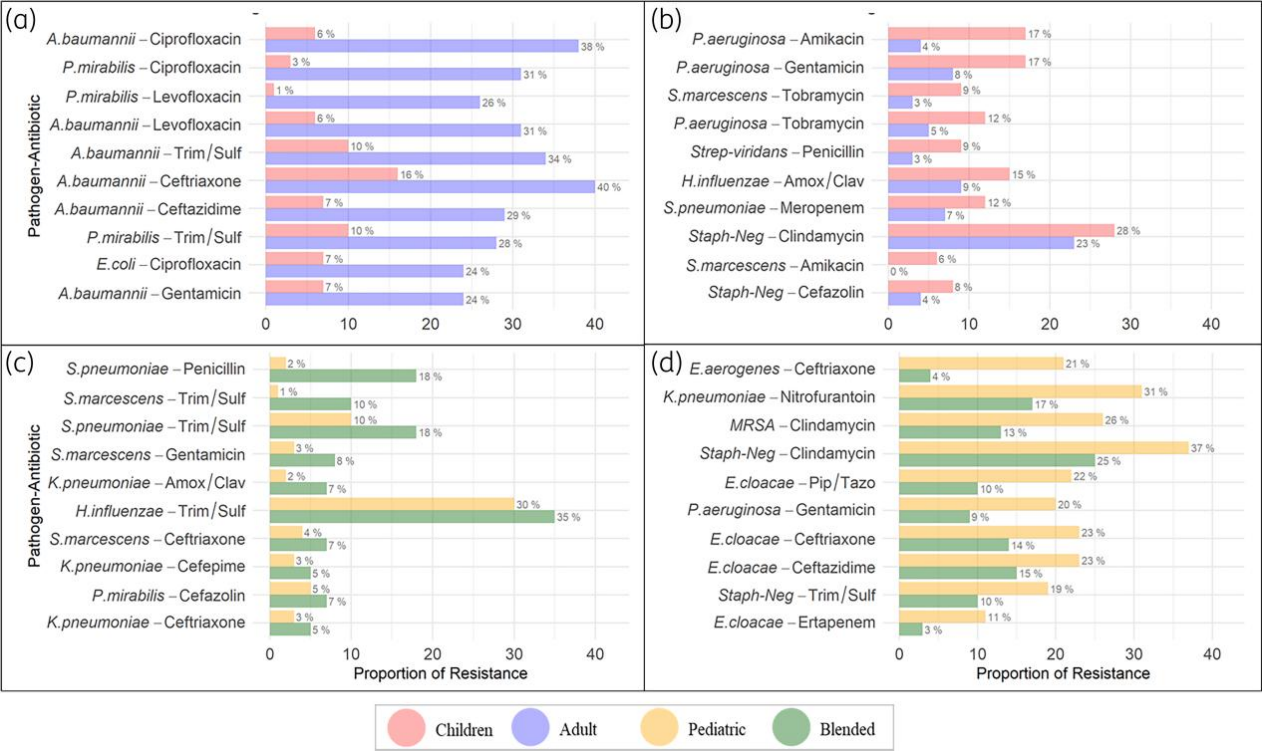
Difference in level of resistance between subgroups. Higher baseline resistance in isolates from: (a) Adults when compared to children. (b) Children when compared to adults. (c) Children in blended facilities when compared to pediatric facilities (d) Children in pediatric facilities when compared to blended facilities.

We compared resistance patterns based on age group (Figure 3a and b) and care setting (Figure 3c and d). The overall difference in the proportion of resistant isolates was higher in adults when compared with children (Figure 3a and b). For example, isolates from adults had a higher proportion of ciprofloxacin-resistant *A. baumannii* (38% versus 6%, *P* < 0.0001) whereas isolates from children had a higher proportion of amikacin-resistant *P. aeruginosa* isolates (17% versus 4%, *P* < 0.0001). Some isolates from children in blended facilities had a higher proportion of penicillin-resistant *S. pneumoniae* (18% versus 2%, *P* < 0.0001) (Figure 3c). However, in general, children treated in paediatric facilities had a higher proportion of resistant isolates when compared with blended facilities (Figure 3d). For example, isolates from children in paediatric facilities had a higher proportion of ceftriaxone-resistant *E. aerogenes* compared with those treated in blended facilities (21% versus 4%, *P* < 0.0001).

### Trend in resistance

**Figure 4.**
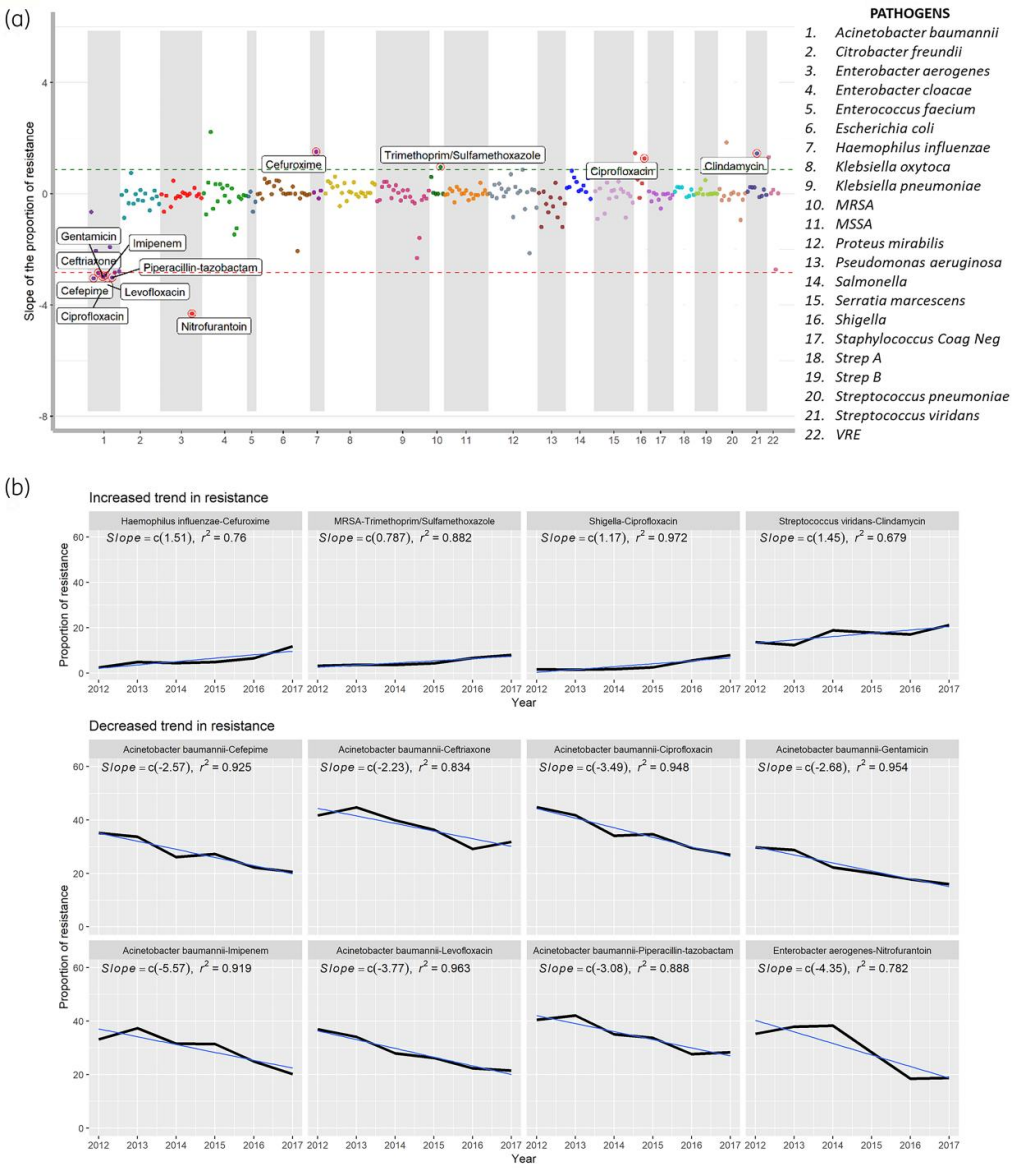
MCS (Multiple Categorical Slope) Plot of the study cohort. (a) MCS plot: Calculated slopes of the antibiotic-resistant-pathogen pairs are plotted on the *Y*-axis, the *X*-axis of the plot shows antibiotics as dots organized by pathogens (listed to the right), in different colored blocks. Statistically significant pairs which increased between 2012-2017 are highlighted and labelled above the upper limit (95th percentile value of the slope) horizonal line while the pairs which decreased are highlighted below the baseline lower limit (5th percentile value of the slope). (b) Patterns of increased/decreased trend in resistance of statistically significant antibiotic-resistant-pathogens.

The MCS plot of the study cohort is shown in Figure 4(a). The predominant pair was ciprofloxacin-resistant *Shigella* sp., which increased from 1.6% to 8% between 2012 and 2017 (slope = 1.17, *R*^2 ^= 0.972, *P* = 0.003) and ceftazidime-resistant *A. baumanii*, which decreased from 34% to 24% (slope = −6.24, *R*^2 ^= 0.981, *P* = 0.004). The positive and negative trends of other statistically significant pairs are depicted in Figure 4(b).

**Figure 5.**
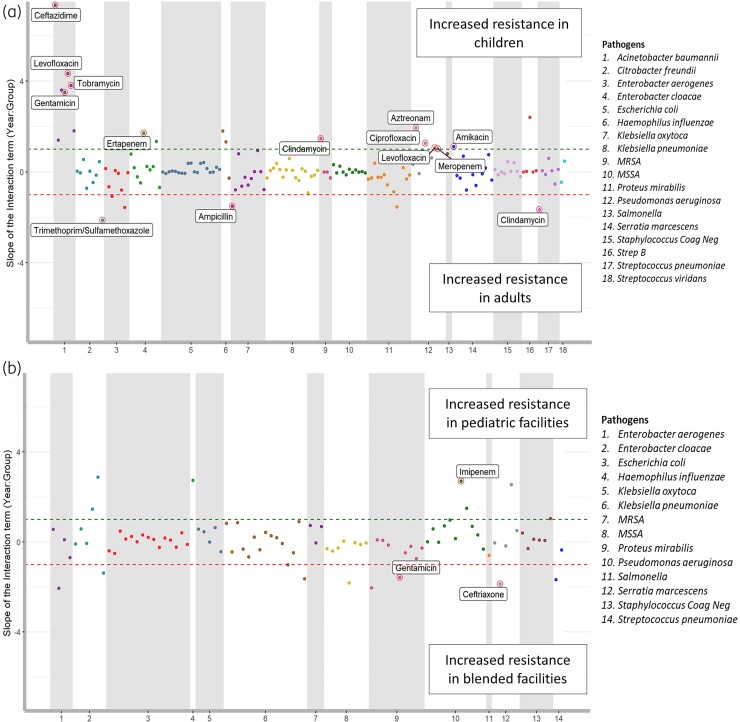
C-MCS (Comparison Multiple Categorical Slope) plot between the subgroups. Only statistically significant changes are highlighted and labelled. (a) Unequal slope of trend in resistance between adults and children (b) Unequal slope of trend in resistance between pediatric and blended facilities.

The pathogen-antibiotic combinations with a significant positive and negative trend for all the four groups are shown in Figure S1. Among isolates from adults, cefuroxime-resistant *H. influenzae* increased from 1.4% to 8% (slope = 1.9, *R*^2 ^= 0.945, *P* = 0.02), whereas ceftazidime-resistant *A. baumanii* decreased from 36.1% to 25.3% (slope = −6.81, *R*^2 ^= 0.972, *P* = 0.004) (Figure S2). Among isolates from children, clindamycin-resistant MRSA increased from 15.5% to 24.2% (slope^ ^=^ ^1.86, *R*^2 ^= 0.992, *P* = 0.0007) whereas nitrofurantoin-resistant *E. aerogenes* decreased from 37.1% to 13.3% (slope = −5.19, *R*^2 ^= 0.959, *P* = 0.009) (Figure S3). The C-MCS plot for adults versus children is shown in Figure 5(a). Ertapenem-resistant *E. cloacae* isolates from children increased significantly compared with adults (children: 0.7% to 9.8%; adults: 2.1% to 2.8%; *P* = 0.00013). In contrast, ampicillin/sulbactam-resistant *Klebsiella oxytoca* increased in adults but decreased in children (adults: 11% to 14%; children: 13% to 7%; *R*^2^ = 0.533) (Figure S4).

In paediatric facilities, ertapenem-resistant *E. cloacae* increased from 0% to 27.1% (slope = 5.16, *R*^2 ^= 0.948, *P* = 0.002) whereas gentamicin-resistant *Proteus mirabilis* decreased from 8% to 0.8% (slope = −1.89, *R*^2 ^= 0.894, *P* = 0.01) (Figure S5a). In blended facilities, cefazolin-resistant *P. mirabilis* increased from 4% to 11% (slope = 1.5, *R*^2 ^= 0.85, *P* = 0.02) whereas nitrofurantoin-resistant *E. cloacae* decreased from 25% to 14% (slope = −1.81, *R*^2 ^= 0.71, *P* = 0.03) (Figure S5b). The C-MCS plot for cultures from children in paediatric versus blended facilities is shown in Figure 5(b). Imipenem-resistant *P. aeruginosa* isolates from children increased in paediatric facilities but decreased in blended facilities (Paediatric: 8% to 14%; Blended: 12% to 10%; *R*^2^ = 0.738). In contrast, ampicillin/sulbactam-resistant *K. oxytoca* increased in blended facilities but decreased in paediatric facilities (Blended: 11% to 12%; Paediatric: 13% to 7%; *R*^2^ = 0.533) (Figure S5c).

## Discussion

We evaluated variation in AR for 22 high-priority pathogens based on age category of patient (child, adult), care setting for paediatric patients and over time. Several statistically significant changes in AR rates were observed between January 2012 and December 2017 as well as over time with respect to age and care setting. Overall, we note a higher level of AR among isolates of the same pathogen for children compared with adults, and children treated in paediatric facilities compared with those treated at blended facilities. However, we also note varying trends for individual bug-drug pairs.

Our findings with respect to the entire HF cohort indicate high resistance rates of Gram-positive pathogens to clindamycin (Figure 2), consistent with a CDC report showing that clindamycin-resistant Group B *Streptococcus* results in 13 000 infections and 720 deaths per year.^38^ Our study also indicated high resistance rates of Gram-negative bacteria to cephalosporins (ceftriaxone, cefuroxime, cefotetan and cefazolin). Emergence of cephalosporin-resistant Gram-negative infections, such as ceftriaxone-resistant *A. baumanii*, third-generation cephalosporin-resistant *Klebsiella* and *E. coli* isolates can have a detrimental impact on clinical outcomes.^39–42^ The emerging trends in resistance rates to clinically relevant antibiotics are worrisome and may lead to the spread of life-threatening infections, especially for inpatient settings. Our study reports an increase in ciprofloxacin-resistant *Shigella* spp. (Figure 4), which is consistent with a health advisory published by the CDC describing an increase in the number of reported cases of ciprofloxacin-resistant shigellosis.^43^

Our study adds to the national concerns about AR as we show differences in resistance patterns between isolates from adults and children. At the baseline level, *A. baumanii* were more resistant in isolates from adults when compared with children (Figure 3a). This was consistent with the literature indicating that carbapenem-resistant *A. baumannii* is increasing in adults. However, in isolates from children, there was a significantly decreased trend of resistance after 2008, which may be related to the expert guidance released by the Infectious Diseases Society of America and the Society for Healthcare Epidemiology in 2007 advising implementation of antimicrobial stewardship programmes in acute care settings to combat MDR *A. baumannii* during this time period.^44–46^ Another finding in our study indicates that *P. aeruginosa* isolates from children were more resistant than those from adults (Figure 3b). This was comparable to a recent analysis of over 87 000 *P. aeruginosa* isolates recovered from US children that showed an increase in MDR *P. aeruginosa* from 15.4% to 26% between 1999 and 2012.^47^  *P. aeruginosa* is known to be especially prevalent among children with cystic fibrosis, up to 80%, but less common among adult cystic fibrosis patients.^48,49^ An example of emerging trend among isolates from adults is cefuroxime-resistant *H. influenzae* (Figure 5a). One possible explanation is the increased selective pressure due to the treatment guidelines for management of community-acquired pneumonia in immunocompetent adults established by the American Thoracic Society and the Infectious Disease Society of America, which recommend cefuroxime for influenza with bacterial superinfection.^50^

We also evaluated variations in resistance patterns based on care setting. For example, we show that *K. pneumoniae* had a higher baseline resistance in isolates from children treated in blended facilities (Figure 3c). *K. pneumoniae* accounts for nearly 15% of all HAIs.^51^ Therefore, it is possible that blended facilities could have a higher proportion of *K. pneumoniae*-associated HAI than paediatric facilities. One key example based on both age and care setting is the emerging trend of ertapenem-resistant *E. cloacae* isolates from children and especially within the paediatric facilities (Figure 5). Ertapenem-resistant Enterobacteriaceae are identified as an important problem associated with an increased 30-day mortality and a significant variation in antibiotic treatment for children with infrequent use of combination therapy.^52^ This rising trend could be due to the emergence and spread of resistant clones, which may be easily transmitted within healthcare settings.^53^

These patterns highlight the growing problem of bacteria developing resistance to first-line therapies segmented by age and the type of care setting. These trends are especially concerning for emergency department providers, because they are often the first point of contact for individuals presenting with these diseases and must make empirical antibiotic selections. Failure to identify and properly treat these organisms can have a devastating impact on patient outcomes.^54^ Although institution-specific antibiograms exist, they are not universally available. One important strategy to combat AR is the use of care setting-specific ASPs based on the type of facility and the age of the patient. ASPs work to promote the appropriate use of antibiotics and to decrease the spread of resistant organisms.^55^ ASPs have optimized antibiotic use and reduced healthcare costs but their effectiveness will be limited until they become more specific to the type of population and care setting.^56,57^ Our work suggests strategies to offer ASPs informed by national and local data. For example, paediatric settings may identify ASP strategies that vary from blended facilities, and blended facilities may consider age-based ASP plans. Many HF data contributors have access to the raw data and can apply the methods described here to develop facility-specific trending for each bug-drug combination.

Our study also identified patterns with negative trends in resistance, including drops in *A. baumanii* resistance in the HF cohort and among adults. The 2019 CDC report on *A. baumanii* indicated that resistance to fluoroquinolones, extended-spectrum β-lactam, ampicillin/sulbactam and trimethoprim/sulfamethoxazole has been decreasing between 2013 and 2017, consistent with the findings of this study.^58^ The increased use of carbapenems as an empirical treatment for *A. baumannii* infections has potentially reduced the selective pressure to develop resistance to other antibiotics.

Our study has known limitations. First, it could not be ascertained whether the infections were community acquired or nosocomial or whether resistance was primary or secondary; the AR rates were generalized to the full study population. Second, despite controlling for the total number of encounters, confounding variables for severity of resistance may exist. However, this does not change our result with respect to the proportion of baseline resistance or trend in resistance. Third, our work is observational and does not provide insights into the drivers for the changes in AR patterns. Although likely to have minor impact, some site-level system configurations, such as suppression of certain results, could also influence the data.

Our study also has several strengths. First, we created the MCS and C-MCS plots, a novel methodology plotting the slope of the proportion of resistance segregated by pathogen-antibiotic combinations to identify significant increase/decrease in the trend in resistance and enabling easy comparison between groups. We were able to discern patterns, identify linear relationships in 302 isolate-antibiotic pairs, repeat the analysis for four groups and focus on significant insights readily apparent in the MCS and C-MCS plots. Second, we used a national data set that combines patient age and facility-level characteristics and validated the accuracy of the HF data source for the first time with internal antibiogram data. Third, we compared the level of resistance and the trend in AR at the care-setting level for the first time.

This study described prevalence and trends of AR among common Gram-positive and Gram-negative bacteria factoring the age of the patient and the care setting. Our visualization methods can inform data-driven ASPs at local, regional and national levels by offering accessible summaries of complex patterns.

## Supplementary Material

dlac128_Supplementary_Data

## Supplementary data

Tables S1 and S2 and Figures S1 to S5 are available as Supplementary data at *JAC* Online.
