# Supplementary material for: Variation in antibiotic resistance patterns for children and adults treated at 166 non-affiliated US facilities using EHR data
Source: JAC Antimicrob Resist. 2023 Jan 2;5(1):dlac128. doi: 10.1093/jacamr/dlac128 (PMC9806600; doi:10.1093/jacamr/dlac128)
Supplement: dlac128_Supplementary_Data [file dlac128_supplementary_data.docx]

**Table S1. HF data validation with CMH antibiogram.** Health Facts (HF) data and Children’s Mercy Hospital (CMH) 2017 antibiogram; MSSA- Methicillin Susceptible *Staphylococcus aureus*; MRSA- Methicillin Resistant *Staphylococcus aureus*;

**Gram-negative pathogens**

**
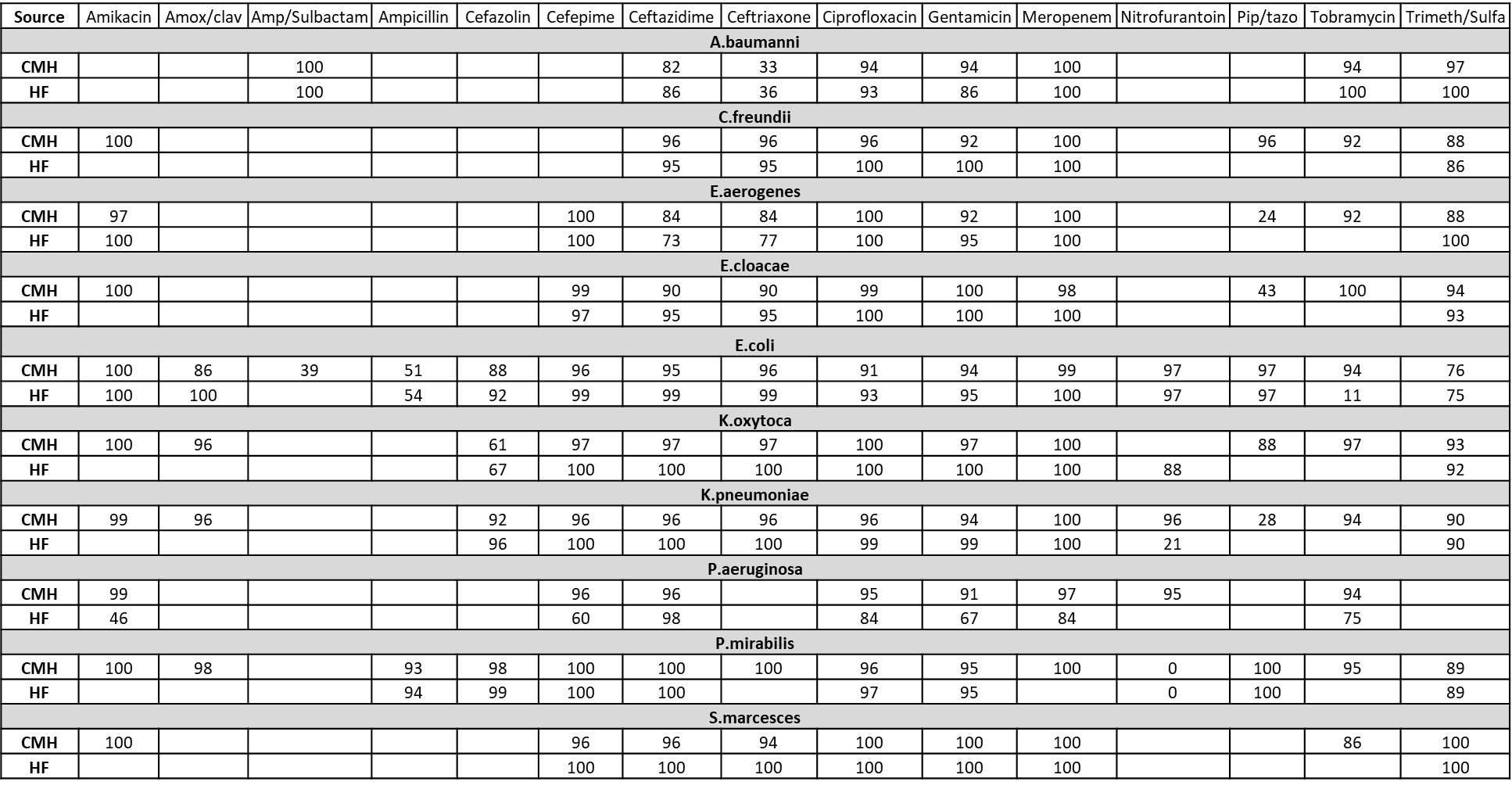
**

**Gram positive Pathogens**

**
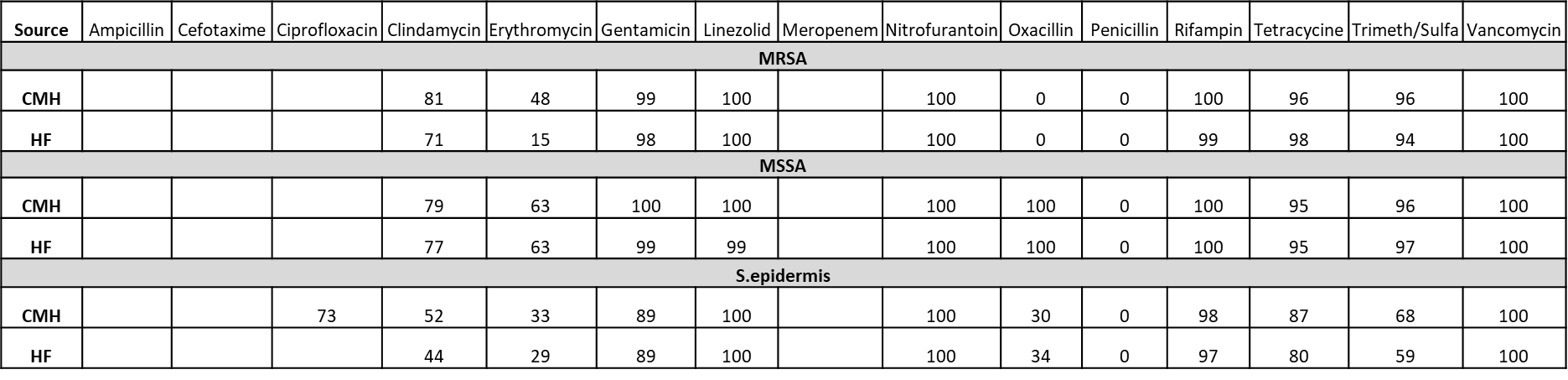
**

**Table S2: Isolate - Antibiotic combinations.** All the clinically relevant isolate-antibiotic combinations that were considered for the study before applying any inclusion criteria.

**
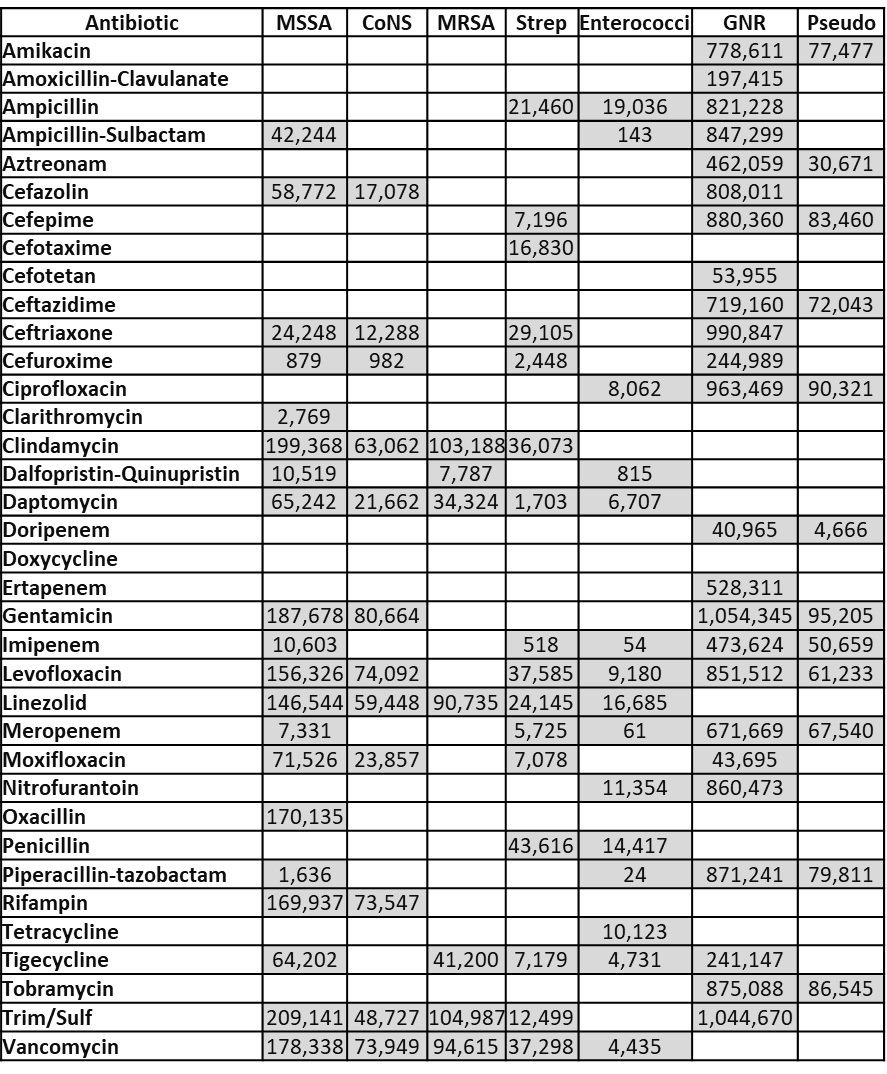
**

MSSA- Methicillin Susceptible *Staphylococcus aureus*; MRSA- Methicillin Resistant *Staphylococcus aureus*; CoNS – Coag Negative *Staphyloccocus*; Strep – *S.pyogenes, S.agalactiae, S.pneumoniae, S.viridans*; Enterococci – *E.faecium*, VRE; Pseudo – *P.aeruginosa*; GNR – Gram negative rod organisms (*C.freundii, E.aerogenes, E.cloacae, E.coli, H.influenzae, K.oxytoca, K.pneumoniae, P.mirabilis, Salmonella spp, S.Marcescens and Shigella spp*).

**Figure S1. The pathogen-antibiotic combinations with a significant positive and negative trend for all the four groups**

**
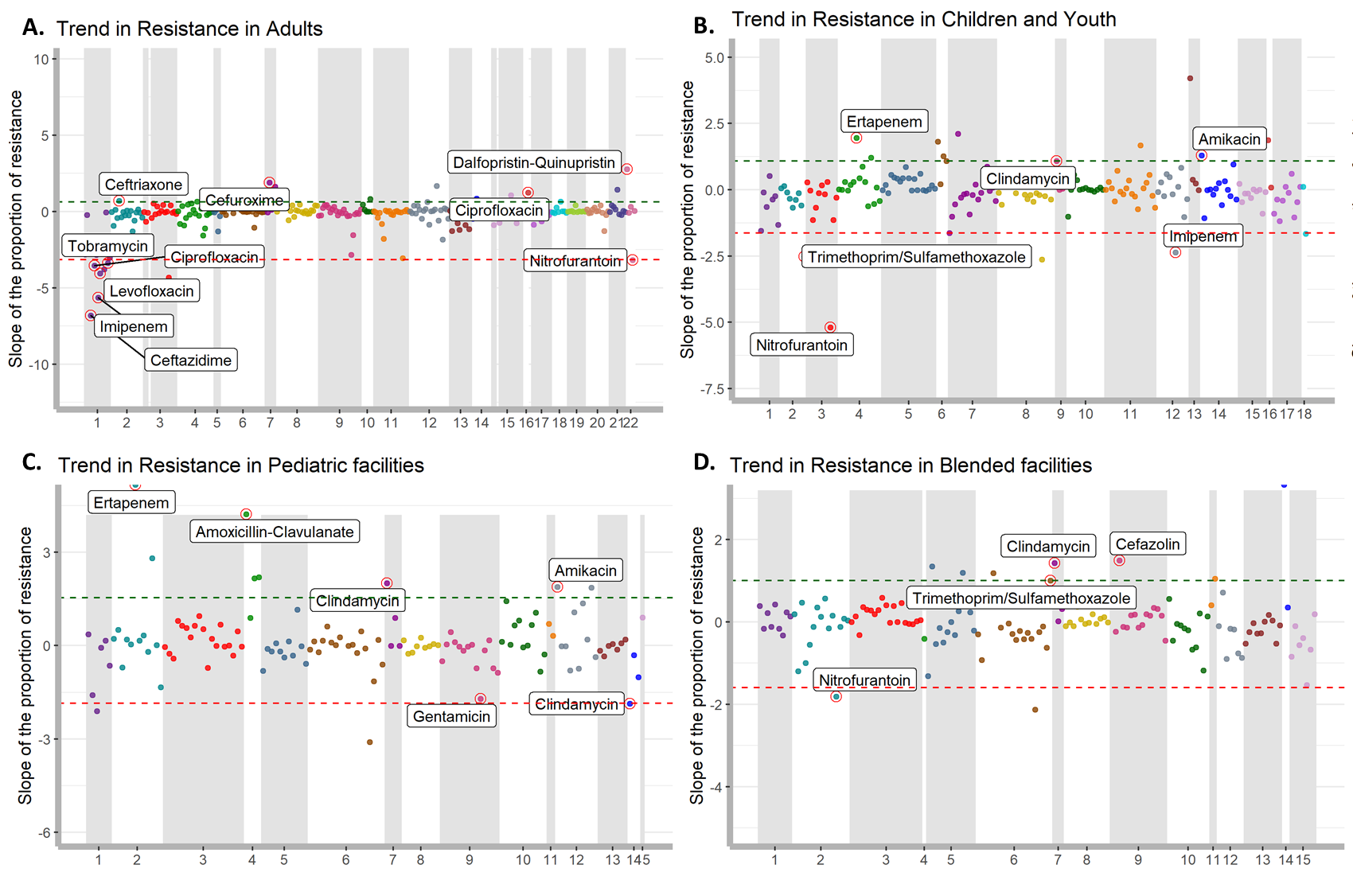
**

**Figure S2. Patterns of increasing and decreasing trend in resistance of statistically significant pathogen-antibiotic isolates from adults.**

**
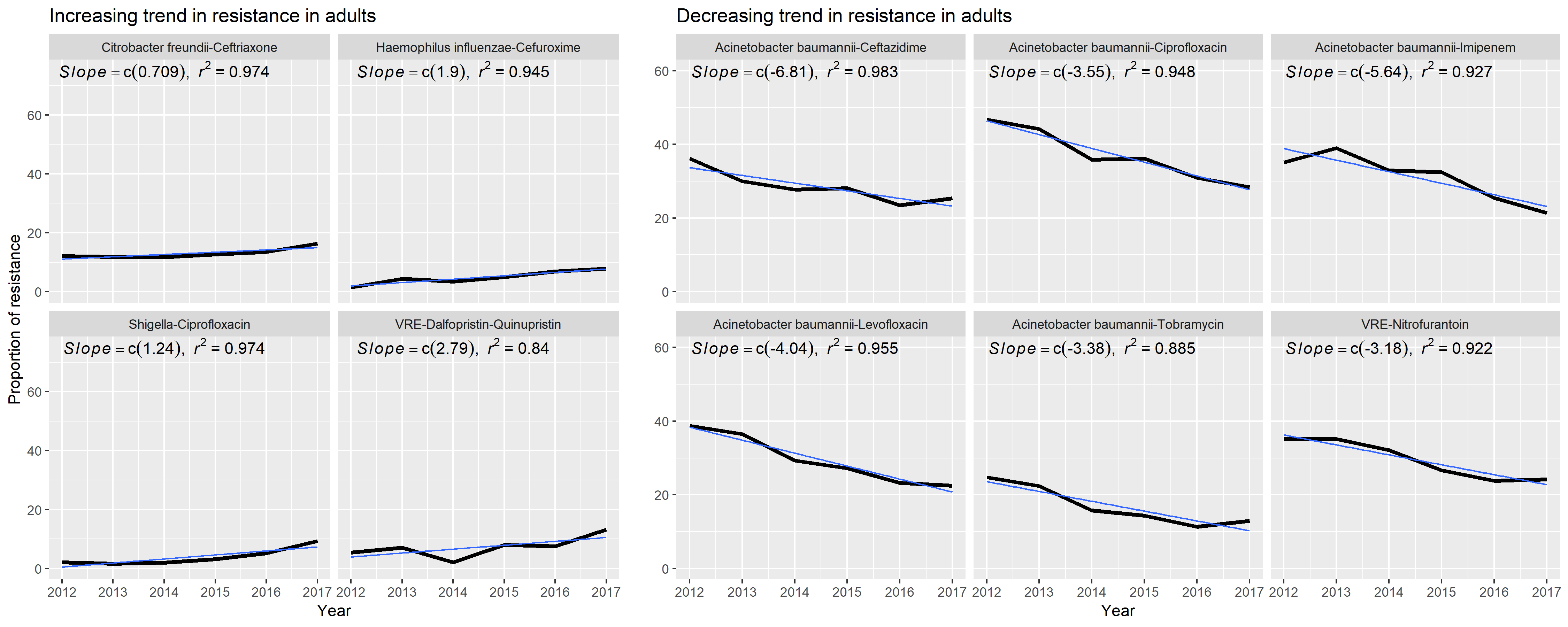
**

**
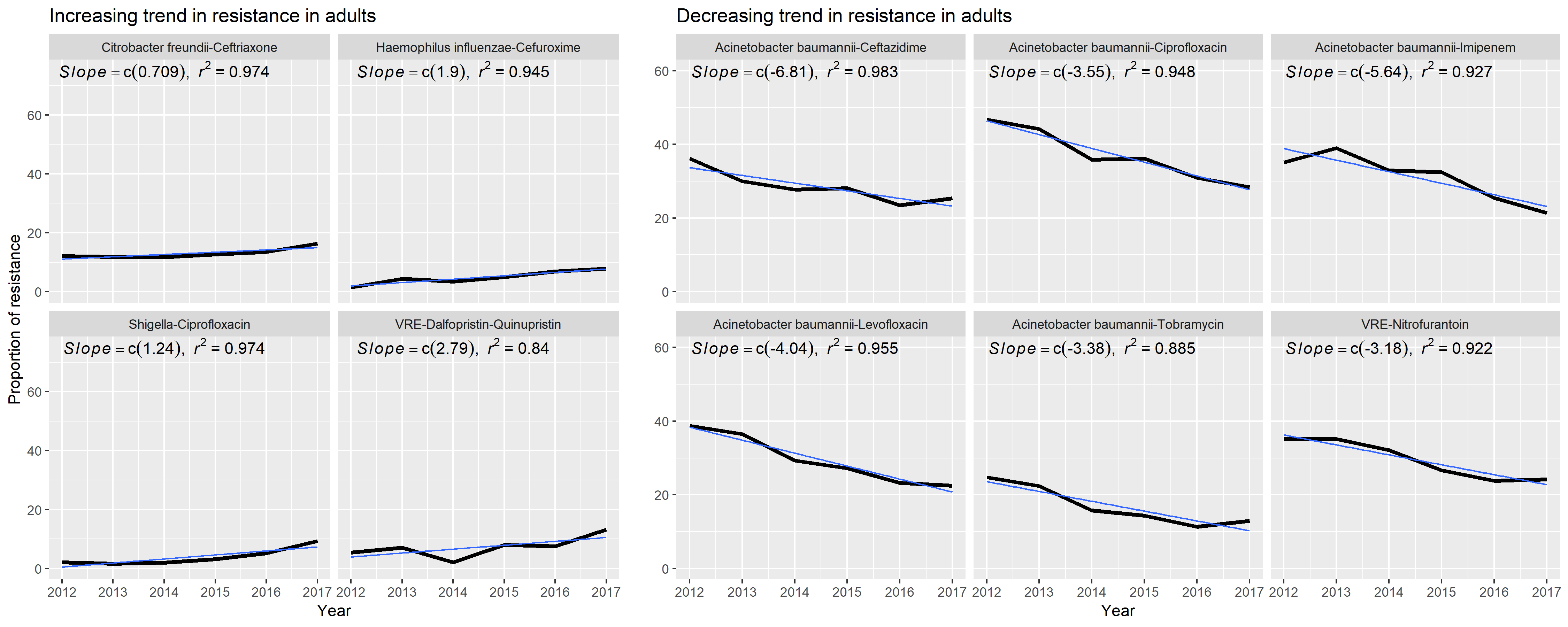
**

**Figure S3. Patterns of increasing and decreasing trend in resistance of statistically significant pathogen-antibiotic isolates from children.**

**
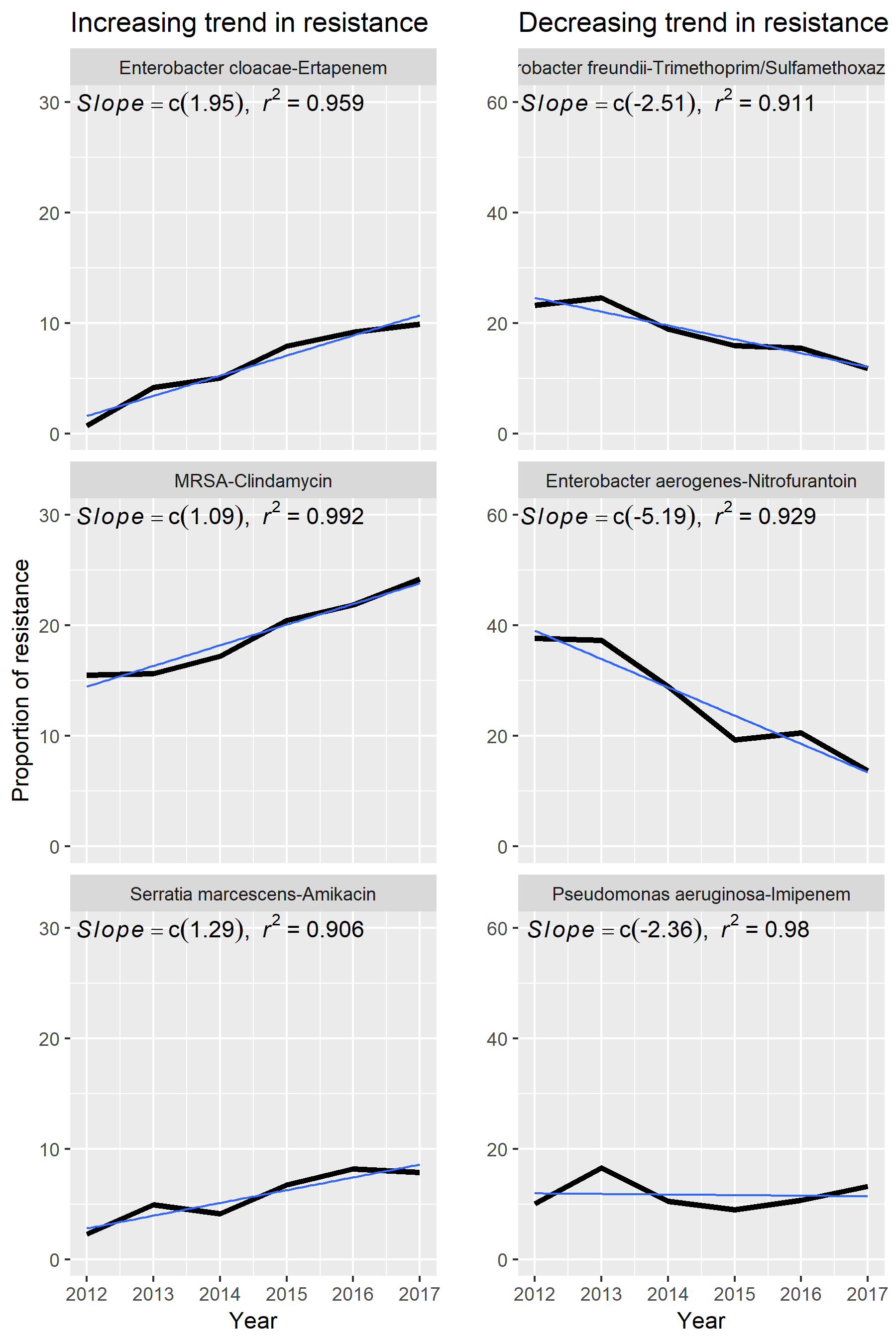
**

**Figure S4. Patterns from C-MCS plot for adults Vs children.** (A) Higher increase in resistance among isolates from adults than children (B) Higher increase in resistance among isolates from children than adults

**
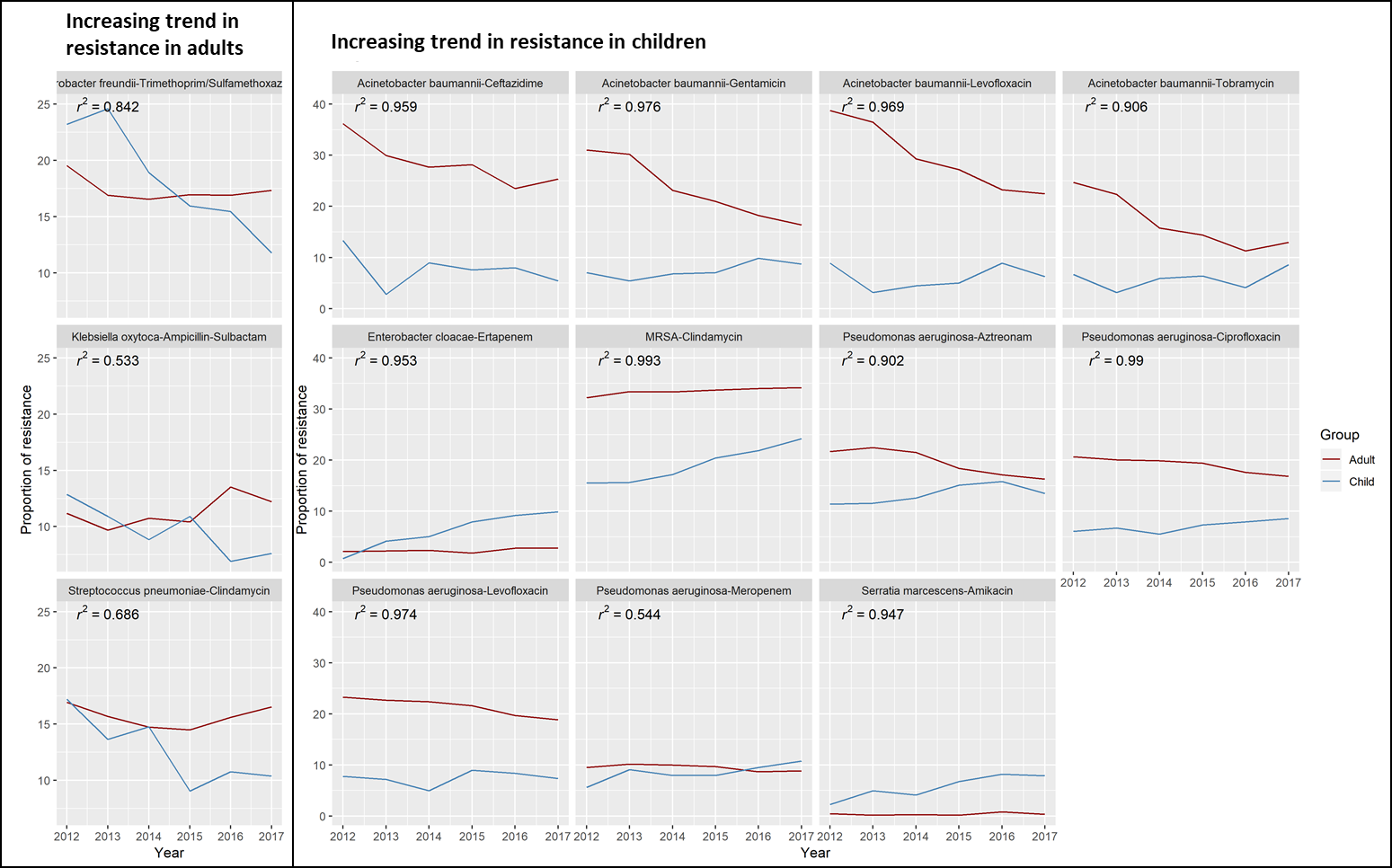
**

**Figure S5. Patterns of trend in resistance among isolates from children by care-setting** (A) Pediatric facilities (B) Blended facilities (C) Patterns from C-MCS plot for children in pediatric vs blended facilities

**A**

**
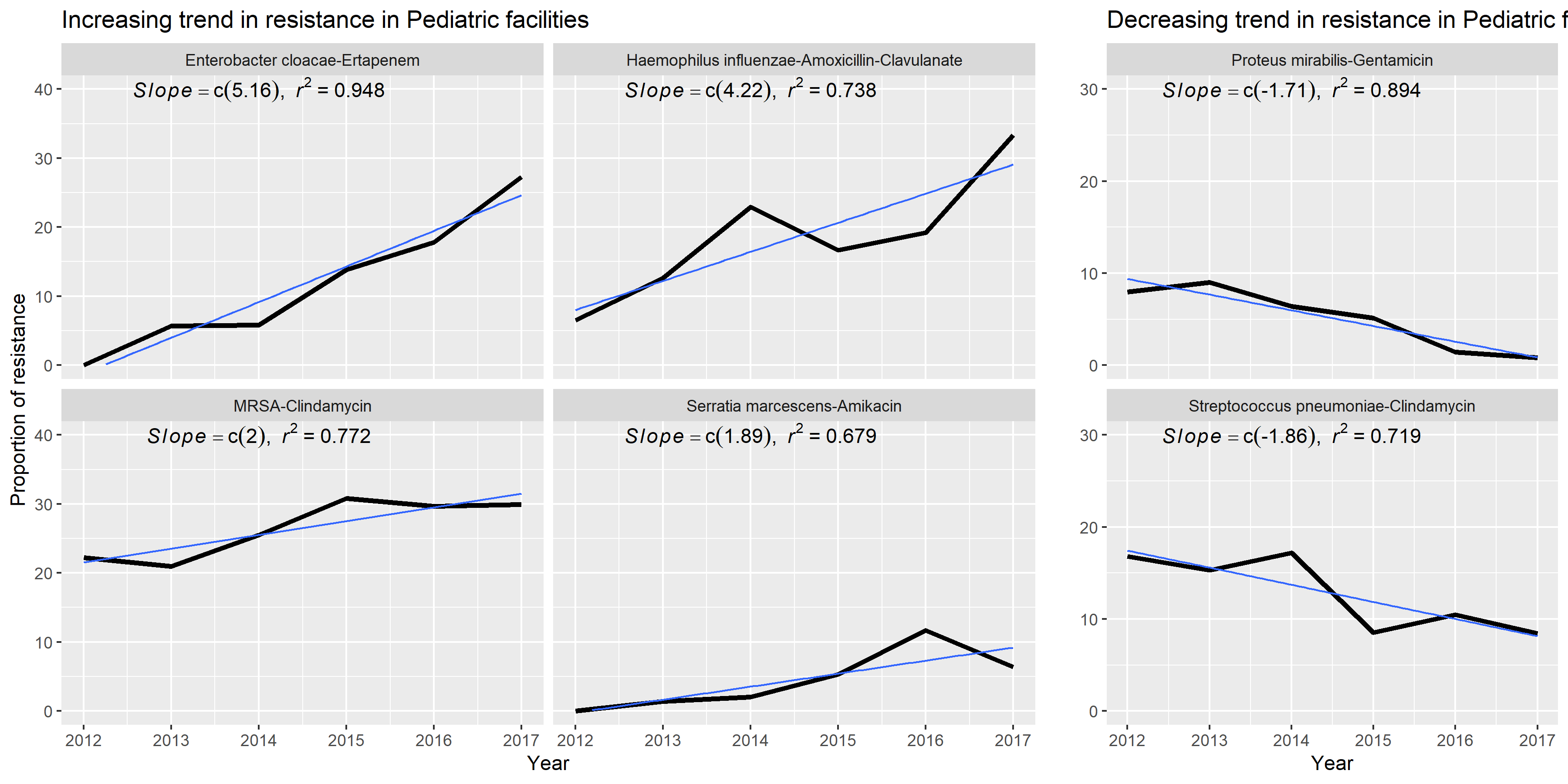
**

**B**

**
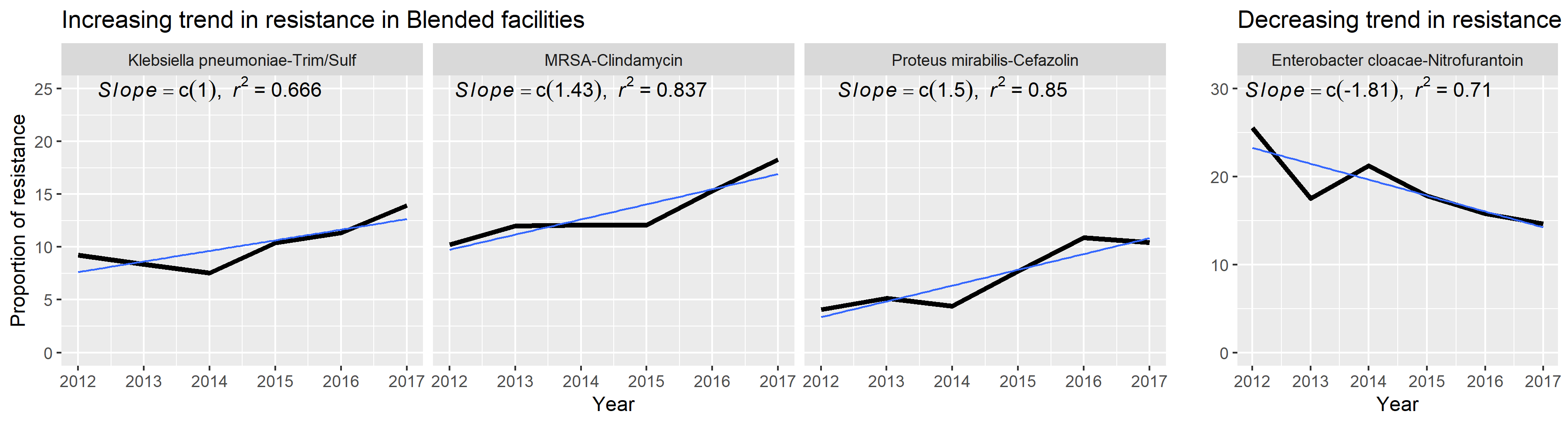
**

**
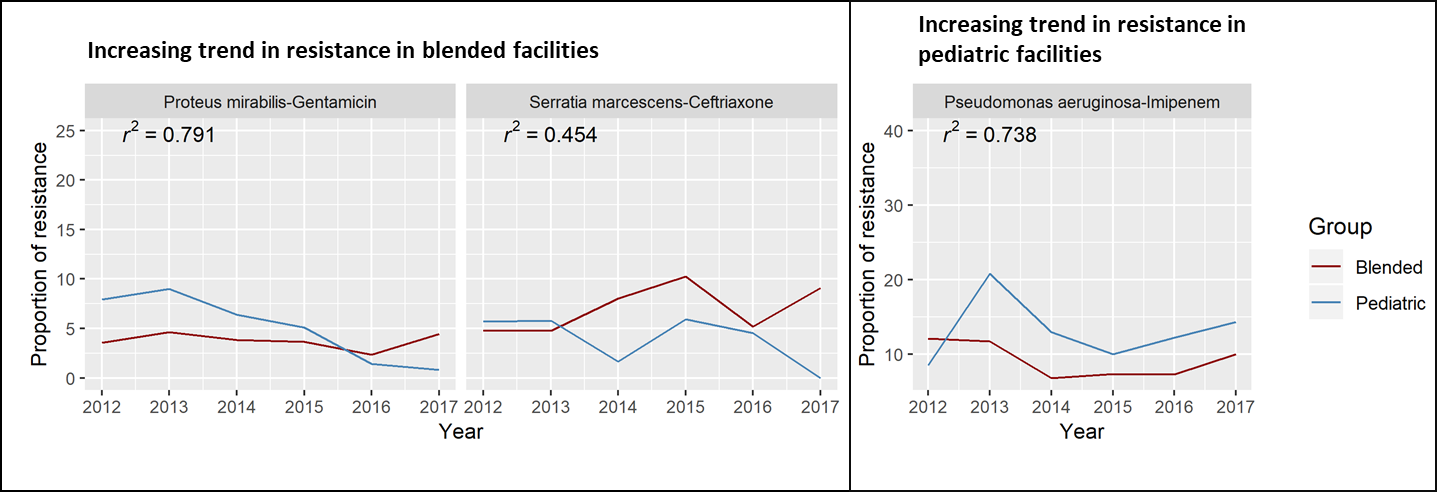
**

**C**
